# How to Vaccinate

**Published:** 1902-01-25

**Authors:** 


### HOW TO VACCINATE.

Commenting on the remarks of Dr. Andrewes concerning susceptibility to re-vaccination, to which we referred last week, Dr. Albert Benthall[1] states that he has recently re-vaccinated a rather larger number of persons—namely, 193 individuals, of whom 162 were males, and 31 were females. Out of the whole number of re-vaccinations there was not one single failure, all the arms developing well-marked vesicles in the three places scarified. Among these cases six had been recently vaccinated with negative result, but they showed no insusceptibility in his hands. In 72·53 per cent. of the cases the vesicles were large and well formed ; in 16·06 per cent. they were moderate in size ; while in just under 11·4 per cent. they were small, but not under a quarter of an inch in diameter. The lymph used was that of the Jenner Institute. The arms were first washed with soap and warm water, and in the case of the men shaved ; the region where vaccination was to take place was then well rubbed with hot boric acid solution (saturated), and afterwards dried with boric lint. The vaccine was placed on the skin in three places, and the epidermis was scratched through the vaccine with a blunt lancet ; hardly any blood was perceptible. In all these cases and many others dealt with in the same manner, the re-vaccination took without undue inflammation or enlargement of glands. The point recommended for vaccination is the outer side of the left forearm. No shield and no lint is advised, the best results being obtained when the following directions are carried out : the undervest sleeve should be turned up and nothing worn under the sleeve of the white shirt which is always very loose. Where a coloured shirt is worn a piece of clean, soft, old cambric handkerchief should be tacked to the inside of the coloured sleeve. In this way the vesicles are hardly touched, and the arm is kept cool. The only application recommended is a liberal and frequent dusting with vinolia powder, and with these precautions it is found as a rule that the vesicles remain dry and unbroken. The lancet was sterilised with boiling water before each separate vaccination.

[1] Lancet, Jan. 18.

